# Advances in Nanotechnology-Based Topical Delivery Systems for Skincare Applications

**DOI:** 10.3390/pharmaceutics18010063

**Published:** 2026-01-03

**Authors:** Ziwei Yan, Sunxin Zhang, Guyuan Wu, Yunxiang Kang, Cong Fu, Zihan Wang, Guoqi Wang, Lu Tang, Wei Wang

**Affiliations:** 1 National Medical Products Administration (NMPA) Key Laboratory for Research and Evaluation of Cosmetics, China Pharmaceutical University, Nanjing 211198, China; 2 State Key Laboratory of Natural Medicines, Department of Pharmaceutics, School of Pharmacy, China Pharmaceutical University, Nanjing 211198, China; 3 Institute for Experimental Molecular Imaging, Uniklinik RWTH Aachen and Helmholtz Institute for Biomedical Engineering, Rheinisch-Westfälische Technische Hochschule (RWTH) Aachen University, 52074 Aachen, Germany

**Keywords:** skincare, nanotechnology, topical delivery technology, skin penetration

## Abstract

The growing demand for effective skincare products that can effectively target specific dermatological concerns has accelerated the development of advanced delivery technologies. Among them, nanocarrier-based topical delivery systems have gained significant attention for their ability to enhance the performance of skincare formulations. Acting as versatile delivery tools, nanocarriers not only stabilize and protect sensitive cosmetic ingredients but also improve their penetration through the skin barrier and enable controlled, sustained, or targeted release. Therefore, this review focuses on the recent achievements of nanocarrier-based topical delivery technology for skincare applications, which systematically summarizes the design principles, mechanisms and functional characteristics of diverse nano-based delivery platforms, including vesicular nanocarriers, lipid-based nanocarriers, emulsion-based nanocarriers, polymeric nanocarriers, inorganic nanoparticles, and inclusion complexes. Meanwhile, these nanocarriers are discussed according to their relevance to the pathogenesis of prevalent skin disorders, highlighting how tailored nanocarriers can address specific therapeutic or cosmetic needs. Overall, this review emphasizes the emerging trends and future perspectives of nanotechnology-based topical delivery systems in modern cosmetology, offering more opportunities for precise, effective and science-driven cosmetic solutions.

## 1. Introduction

Physiologically, the skin is organized into three primary layers—the epidermis, dermis, and subcutaneous tissue—which collectively provide barrier protection, secretion, excretion, and selective absorption [1]. The outermost epidermis, mainly composed of keratinocytes, forms the critical stratum corneum (SC) barrier. Beneath it, the dermis supplies structural support, nourishment, and innervation, while the subcutaneous layer serves as an insulating and cushioning adipose depot [2,3,4]. Additionally, secretions from sweat and sebaceous glands contribute to the surface microecological balance and lipid film, which influence the percutaneous absorption of external substances [5].

Nanocarriers are a class of nano-dispersed systems formed by natural or synthetic polymers and inorganic nanomaterials [6,7,8]. Their particle sizes are usually in the range of 10 to 1000 nanometers and have strong biofilm penetration ability and highly efficient material delivery properties [9,10,11]. In skincare, nanocarrier-based topical technology significantly enhances the permeability and bioavailability of active ingredients across the SC. By encapsulating actives within liposomes, solid lipid nanoparticles (SLNs), nanoemulsions, etc., nanocarriers protect them from degradation and improve stability [12,13,14]. Their mechanisms include reducing particle size, increasing carrier deformability, disrupting SC organization, modulating surface charge, and optimizing particle dimensions, thereby promoting deeper skin penetration and efficacy [15,16,17]. Skin is also an active immune and microbial organ, and thus, effective delivery strategies should ultimately be compatible with these biological systems. Meanwhile, skin microbiome is of great importance for barrier health, and ideal nanocarrier systems should be formulated to support, or at least not disrupt, this ecological balance. In addition, nanocarrier-based technology enables precise modulation of active ingredient concentrations, carrier architectures, and formulation ratios so as to meet the personalized needs of consumers regarding cosmetic efficacy, texture, and skin feel. Such personalized customization not only improves the relevance and applicability of cosmetics but also creates new opportunities for market segmentation and differentiated competition within the cosmetics industry [18,19,20,21,22,23,24]. However, the safety of nanocarriers and their corresponding regulatory measures have become focal points in both academia and the industry. Currently, there are remaining critical gaps in the regulatory landscape, including a lack of globally harmonized definitions, insufficient long-term safety data for many nanomaterials, and the absence of specific guidelines for novel and complex nanocarrier systems, underscoring the urgent need for more adaptive and comprehensive oversight. At the same time, although nanocarrier-based technologies offer remarkable advantages in improving the efficacy and bioavailability of cosmetic ingredients [25,26], they also raise unavoidable safety and environmental issues. For example, certain nanocarriers may cause skin sensitization, irritation, or allergic reactions due to their small particle size and high surface reactivity [27,28,29]. In addition to direct biological risks, the production, utilization, and disposal of nanomaterials may also contribute to environmental pollution and ecological toxicity, posing new challenges to sustainable manufacturing and waste management. Therefore, in order to address the safety issue of nanocarriers, governments and regulatory agencies worldwide have established or are developing rigorous frameworks to evaluate and monitor the safety of nanocarriers used in cosmetic products [30,31,32]. In parallel with these regulatory efforts, the cosmetic industry and scientific community are actively exploring green synthesis methods, biodegradable carriers, and alternative natural nanomaterials to minimize toxicity and environmental impact. Collectively, these initiatives aim to ensure the safe, responsible, and sustainable application of nanocarriers in cosmetic formulations.

The literature search for this review was conducted primarily using the PubMed, Web of Science, and Scopus databases. Our search strategy employed a combination of key terms and their variants, including “nanocarrier”, “topical delivery,” “cosmetic/skincare,” “liposome,” “solid lipid nanoparticle,” and “skin penetration.” To ensure the inclusion of both foundational and contemporary advances, the search was focused on articles published within the past 5 years. We prioritized peer-reviewed original research and authoritative reviews that provided clear mechanistic insights into nanocarrier–skin interactions or demonstrated definitive applicational strategies for addressing specific cosmetic skin concerns, such as hydration, anti-aging, and hyperpigmentation.

In summary, this review provides a comprehensive overview of nanocarrier-based topical delivery systems and their transformative role in cosmetic areas. This article emphasizes how nanotechnology enhances the permeability, stability, and bioavailability of active ingredients, thereby improving efficacy and enabling personalized skincare solutions, while highlighting its uniqueness by systematically linking the design of nanocarriers with practical strategies for addressing specific skin issues such as acne, aging, and pigmentation. Furthermore, unlike earlier reviews, we critically examine the persistent gaps in current regulatory frameworks for nanomaterials and offer forward-looking insights into future breakthrough directions in this field. By integrating material innovation with formulation science, safety assessment, and regulatory foresight, this review not only clarifies the state of the art but also equips researchers and industry professionals with an actionable and forward-looking perspective to advance the next generation of nanocosmetic products.

## 2. Nanocarrier-Based Topical Delivery Systems: Current Class and Cosmetic Benefits

The topical delivery nanocarriers employed in cosmetics primarily encompass vesicular nanocarriers, lipid-based nanocarriers [33], emulsion-based nanocarriers, polymeric nanocarriers, inorganic nanoparticles [34], and inclusion complexes [35] (Table 1). Among the key physicochemical parameters defining these nanocarriers, particle size plays a vital role, serving as a fundamental determinant underlying their unique biological effects [36]. Extensive research has demonstrated that nanocarriers with particle sizes typically ranging from 10 to 1000 nm exhibit markedly distinct physicochemical properties [37], such as altered saturation solubility, dissolution kinetics, hydrophilic–hydrophobic balance, and structural stability, as well as modified biological characteristics [38,39,40]. These size-dependent variations significantly impact the absorption, distribution, metabolism, and excretion profiles of the active ingredients [41], thereby contributing to enhanced dermal and topical permeation, improved bioadhesion and internal stability [42], targeting capabilities [43], controlled or sustained release properties [44,45,46], and increased cellular internalization efficiency [47,48,49]. Besides the particle size, encapsulation efficiency (EE), defined as the percentage of the total initial active ingredient successfully incorporated into the nanocarrier system, stands as a pivotal physicochemical parameter in the development and evaluation of topical delivery systems. Together with the closely related parameter of drug loading, EE plays a decisive role in determining the practical applicability, cost-effectiveness, and eventual commercial feasibility of a nano-formulation [50,51,52,53].

## 3. Nanocarrier-Based Topical Delivery Technology for Skincare

In recent years, with social progress and an increased awareness of personal health, public concern for skin care has increased significantly. A series of skin issues, such as natural skin aging, pigmentation-related spots and dullness, frequent acne, hair loss, skin allergies, dryness, and lack of moisture, have become common sources of distress in many people’s daily lives. With the rapid development of modern technology and the expanding cosmetic market, nanocarrier-based topical delivery technology has emerged as a highly prospective and efficient approach in the field of skincare [67]. Crucially, the efficacy of this technology fundamentally relies on nanocarriers, which serve as the versatile delivery platforms as systematically categorized in the preceding section [68]. By utilizing unique physical or chemical methods, topical delivery technology can precisely deliver active ingredients to the cuticle or even the dermis of the skin, thereby achieving root-cause treatment of skin issues and injecting new vitality and hope into the field of skincare [69]. Whether it is to ameliorate skin aging, reduce pigmentation, treat acne, or alleviate skin sensitivity, topical delivery technology ensures optimal absorption of active ingredients for targeted and effective skincare [70].

Given this foundation, we have carefully organized and illustrated the main mechanisms of these common skin issues, intending to help readers gain a clear and more accessible understanding of their root causes. Through this effort, we aim to provide a solid foundation for future research on targeted treatments, encouraging researchers to develop more precise and effective therapeutic strategies. By clarifying the pathogenic mechanisms of common skin issues, this review seeks to facilitate the development of targeted interventions that enable individuals to effectively tackle and alleviate these concerns, ultimately restoring skin health and confidence.

### 3.1. Anti-Aging

Skin aging is a natural process determined by genetic factors and influenced by various environmental factors, including intrinsic aging and extrinsic aging [71]. Intrinsic aging is a gradual, genetically determined process associated with physiological changes over time, while extrinsic aging results from chronic exposure to environmental stressors. Among these external influences, repeated exposure to ultraviolet (UV) light from sunlight over a long period of time is an important environmental factor contributing to skin aging, and this phenomenon is also referred to as photoaging [72]. Cyclobutane pyrimidine dimers (CPDs) are DNA lesions formed by the covalent bonding of adjacent pyrimidine bases, primarily due to UV light exposure, and are significant contributors to mutagenesis and skin cancer risk. In addition to UV radiation, other environmental factors such as pollution, smoking, poor nutrition, and oxidative stress also contribute to the progression of extrinsic aging, further compounding the decline in skin barrier function and overall skin health.

Fortunately, as scientific research continues to advance, researchers have successfully discovered several active ingredients with significant anti-aging potential. These ingredients not only assist in scavenging excess free radicals and mitigating oxidative stress within the body but also combat the damage caused by non-enzymatic glycation (NEG) reactions and photoaging [73]. However, there are numerous challenges in practical applications. For instance, vitamin C is prone to oxidation and instability, retinol can cause skin irritation and photosensitivity, and astaxanthin exhibits photosensitivity and insufficient stability. These limitations hinder their ability to deeply penetrate the skin and exert their anti-aging effects [74,75,76]. To overcome these drawbacks, researchers have developed various nanocarrier-based delivery systems as transport vehicles for active ingredients, which aim to enhance the bioavailability and skin permeation of anti-aging components [77]. By optimizing the stability and penetration of the ingredients, these nanocarriers can precisely deliver the core components to deeper layers of the skin, thereby achieving more effective anti-aging outcomes [78,79].

Tansathien et al. prepared Porcine placenta extract (PPE)-containing polymeric nanoparticles using thin-film hydration and probe sonication methods. Compared to the control group, these drug-loaded nanoparticles reduced reactive oxygen species (ROS) generation from 180.58 ± 9.15% to 89.90 ± 19.30% in epidermal keratinocytes, significantly alleviating skin inflammation and thereby slowing down the aging process [80]. Algahtani et al. encapsulated Retinol palmitate (RT) into a nanoemulsion and further incorporated it into a hydrogel system to form a nanoemulsion gel (RT-NEG) and showed that the RT-NEG system had more than 4-fold drug deposition rate and more than 2-fold skin penetration compared to RT emulsion and RT gel, indicating that this delivery system has great potential for application against skin aging [81]. Joo et al. developed a polymeric micellar formulation using insoluble oleanolic acid as the main wrinkle-reducing ingredient in cosmetic products, and their clinical results showed that skin roughness decreased by 0.673% and 7.835% after 4 and 8 weeks of use, respectively, and skin wrinkles were significantly improved as well [82].

### 3.2. Skin Whitening

Melanocytes, located in the basal layer of the skin, primarily produce melanin granules [83]. In response to factors such as UV radiation and endocrine imbalances, the activity of melanocytes in the skin increases, leading to an elevation in melanin production [84]. Furthermore, melanocytes absorb the colorless amino acid tyrosine, which, under the influence of tyrosine hydroxylase, is converted into dopa and subsequently oxidized into melanin. As melanin accumulates, it condenses into melanin granules that are stored within the dendritic processes of melanocytes. These granules are then phagocytosed by epidermal basal cells, causing the epidermal cells to transition from being colorless to pigmented. As these cells migrate towards the epidermal layer, the melanin granules eventually emerge and deposit in the SC, manifesting as darker spots or patches compared to normal skin tone, known as pigmented spots or macules [85,86,87]. The fundamental mechanisms of the active ingredients in skin-lightening and spot-removing cosmetics are primarily embodied in four aspects (Table 2).

The efficacy of spot-removing and whitening cosmetics primarily relies on the synergistic effects of one or multiple active ingredients, yet their direct application is fraught with numerous drawbacks and limitations. For instance, compounds such as acetophenone, glabridin, and niacinamide may elicit adverse reactions, including skin irritation, allergies, redness, swelling, and itching. Arbutin may cause skin irritation and melanin deposition, vitamin C and its derivatives (e.g., magnesium ascorbyl phosphate) suffer from poor stability and limited absorption, and titanium dioxide can lead to skin dryness, allergies, and other undesirable reactions [94,97,98,99,100]. Hence, the application of topical delivery technology to address the deficiencies of active ingredients with whitening and spot-removing properties and to enable their precise delivery to targeted skin layers can ensure maximum efficacy of these ingredients in the deeper layers of the skin. This approach effectively inhibits melanin formation, fades existing pigmentation, and significantly enhances the overall effectiveness of skin-lightening and spot-removing treatments.

Li et al. developed a chitosan-based polymeric nanoparticle co-encapsulating Epigallocatechin gallate (EGCG) and Ferulic acid (FA) and modified with genipin. These nanoparticles, with high FA/EGCG loading, strongly inhibited tyrosinase activity and reduced the relative melanin content in treated cells by 63.2 ± 0.4% compared to other groups [101]. Sheweita et al. formulated a nanoemulsion primarily consisting of Trans-Resveratrol (T-Res), which, compared to control groups, effectively regulated the expression of melanogenic proteins, resulting in a more than three-fold reduction in skin melanin synthesis and a notable decrease in pigmentation density [102]. Hsieh et al. prepared catechin-loaded transferosomes, and in vitro skin penetration experiments demonstrated an 85% increase in skin permeability of the transfer agent compared to an aqueous catechin solution, and in vivo skin whitening experiments demonstrated that the transfer agent had a favorable tyrosinase inhibitory effect [103].

### 3.3. Anti-Acne and Oil Control

“Acne”, commonly known as “teenage acne” or “Acne Vulgaris”, is a prevalent chronic inflammatory skin condition characterized by various manifestations such as comedones, papules, pustules, nodules, cysts, and scars, and is often accompanied by excess sebum secretion [104]. The pathogenesis of acne is multifactorial, involving several key mechanisms. For instance, adolescents experience androgen dysregulation due to physical development, leading to excessive androgen secretion that stimulates sebaceous glands to overproduce sebum. Abnormal affinity of sebaceous gland androgen receptors further exacerbates sebum hypersecretion. Excess sebum, combined with desquamated epidermal debris, clogs pores and initiates acne development. Abnormal keratinization of follicular keratinocytes narrows follicle openings, trapping sebum and forming comedones. Additionally, follicular overgrowth of Cutibacterium acnes triggers inflammatory responses, driving acne progression [105,106,107,108]. Propionibacterium acnes can hydrolyze triglycerides in sebum to produce free fatty acids, which stimulate inflammatory reactions in and around hair follicles.

The diversity of acne vulgaris undoubtedly complicates its treatment and management, making it crucial to select products tailored to individual skin conditions and acne types from the wide array of available medications and cosmetic formulations. However, these ingredients, due to their inherent physicochemical properties or improper usage, may potentially cause adverse skin reactions, including but not limited to allergies and peeling, and even exacerbate acne conditions, thereby imposing an additional burden on patients’ skin health. In light of the current challenges, researchers are actively exploring the use of topical delivery technologies to overcome the limitations associated with the application of acne-fighting active ingredients [109]. This technology aims to ensure optimal efficacy within the SC and dermis, thereby more effectively reducing inflammation, regulating sebum secretion, cleansing and unclogging pores, and enhancing the effectiveness of cosmetics in treating acne [110].

Le et al. developed a Curcumin (CUR)-loaded nanoemulsion, which showed potential antimicrobial activity against *Staphylococcus aureus* and *Escherichia coli*, exhibiting the maximum zones of inhibition (21.00 mm and 18.4 mm, respectively) as compared to the positive control group [111]. Amer et al. developed vitamin C-based nanovesicles encapsulating quercetin, and the experimental results showed that the mean zone of inhibition was 15.00 ± 1.23 mm, and the drug-carrying formulation group demonstrated good anti-acne activity. The results of human experiments showed that the application of the drug-carrying formulation group significantly reduced the inflammatory lesions (77.90%), non-inflammatory acne (11.80%), and total lesions (55.30%) [112]. Leignadier et al. used a dendritic macromolecule consisting of 48 lysine residues (G2-dendritic polymer) in an anti-acne study and showed that the G2 dendritic macromolecule significantly reduced the inflammatory factor IL-8, which is associated with acne, through its three-dimensional structure. Meanwhile, it did not affect the non-acne-causing bacteria and the cutaneous commensal bacteria but positively impacted the commensal bacterial survival instead, thus decreasing the number of acne outbreaks [113].

### 3.4. Hair Loss Prevention or Treatment

The phenomenon of hair loss varies according to different factors and types, and the physiological mechanisms behind it are quite complex, involving many aspects such as genetics, hormones, environment, nutrition, and medications [114,115]. Currently, the only medications approved by the United States Food and Drug Administration (FDA) for the treatment of hair loss are topical minoxidil and finasteride. Minoxidil, when used to treat hair loss, is converted into its active metabolite minoxidil sulfate, which functions as a potent arteriolar vasodilator. It activates potassium channels on peripheral arterial smooth muscle and induces cellular proliferation. However, its side effects can include contact dermatitis, headache, and hirsutism. On the other hand, finasteride works by inhibiting the enzyme 5-α-reductase, thereby blocking the conversion of testosterone into its active form, dihydrotestosterone, which is implicated in hair loss. While effective in treating hair loss, finasteride can also cause side effects such as scalp itching, burning sensations, contact dermatitis, and erythema [116,117,118].

Despite the hope offered by FDA-approved medications for hair loss, their limitations and potential drawbacks have spurred researchers to explore novel therapeutic avenues. In recent years, topical delivery technology has emerged as an innovative drug administration method, demonstrating remarkable advantages and potential in the field of hair loss treatment. This technology enables direct penetration of active drug ingredients through the skin to the hair follicles and scalp tissue, significantly enhancing permeation efficiency and bypassing the first-pass effect, thereby improving bioavailability. Topical delivery technology boasts numerous strengths, including high safety, targeted delivery, convenient administration, and applicability to various types of hair loss. By improving the nutritional supply and growth environment of hair follicles, this technology effectively promotes hair regrowth, offering an efficient and convenient treatment option for patients suffering from hair loss [119].

Salimi et al. designed quercetin-loaded SLNs to protect human hair from UV-B light in vitro. Based on the obtained results, the quercetin-containing SLNs controlled peak-to-valley roughness and reduced chemical changes and protein loss, thereby effectively preventing the damage to hair caused by UV-B radiation [120].

### 3.5. Skin Soothing and Repairing

Skin allergy syndrome is a complex cutaneous disorder characterized by hypersensitivity to mild irritants, presenting with symptoms such as tingling, itching, burning sensations, erythema, and tightness. This condition may be caused by genetic factors, gender, age, hormone levels, psychiatric factors, physical and chemical factors, lifestyle, and secondary association with other skin conditions [121,122,123,124]. Currently, the first-line medications for skin sensitivity include antihistamines, glucocorticoids, immunosuppressants, and cooling/itching relief agents [125,126,127]. In the realm of skincare, targeting skin sensitivity and plant extracts are predominantly utilized, often in synergy with moisturizing ingredients. Examples include *Centella asiatica* extract, chamomile extract, and aloe vera extract, which not only soothe the skin but also effectively reduce inflammation and repair damaged skin barriers, providing comprehensive care for sensitive skin [128,129,130,131]. Despite their expected benefits, these ingredients can also be one of the primary triggers for skin sensitivity. In addition, the presence of a number of potential irritants can exacerbate the clinical manifestations of sensitive skin symptoms [132]. Consequently, apart from banning the addition of irritants in cosmetics, the use of topical delivery systems to encapsulate soothing active ingredients can help minimize their potential irritation and offer a safer and more effective skincare option for individuals with sensitive skin. For instance, So et al. developed a novel nanomaterial termed treated *Gelidium amansii* (Ga) cellulose nanocrystal (TGa CNC), derived from Ga, an edible seaweed. Their study demonstrated that TGa CNC effectively modulated UVB-induced phosphorylation and translocation of c-Jun, a major component of AP-1 transcription factor complex, in HaCaT cells [133].

### 3.6. Skin Moisturizing

The physiological mechanisms of skin dehydration primarily encompass the natural evaporation of water and the skin’s ability to retain moisture, particularly mediated by the functional integrity of its structure, especially the SC. When the rate of water evaporation exceeds the skin’s internal replenishment, skin dehydration occurs. Additionally, the SC serves as a natural barrier, and its health status directly impacts the skin’s ability to retain moisture [134,135,136]. To maintain optimal skin hydration, it is crucial to adopt appropriate moisturizing measures while avoiding disruption of the skin’s structure.

Upon understanding the mechanisms of skin dryness or dehydration, researchers have embarked on exploring effective ways to replenish skin hydration through skincare products. These products typically contain specific hydrating ingredients and formulations that work by enhancing the skin’s ability to maintain moisture, repairing damaged skin barriers, or providing immediate hydration to help restore and maintain the skin’s supple and hydrated state. Making use of these products can address the problems of skin dehydration effectively and promote overall skin health and vitality.

Ni et al. designed hyaluronic acid (HA)-modified Undecylenoyl phenylalanine (UP) liposomes (HA-UP-LPs) to enhance skin permeability, and their results showed that the skin retention of UP in the HA-UP-LPs formulation group was increased compared to that of the free UP formulation group over 24 h, and the skin permeability of UP was also increased. In addition, low molecular weight HA (LMW-HA, especially 5 kDa and 8 kDa) could promote the entry of large amounts of water into the SC, epidermis, and even the dermis [137].

All in all, based on the distinct pathophysiological characteristics of various skin concerns, different nanocarriers demonstrate differentiated clinical advantages. Lipid-based carriers (e.g., SLNs/NLCs, nanoemulsions), leveraging their film-forming ability and skin lipid affinity, show marked efficacy in moisturization and barrier repair [138]. Polymeric nanoparticles and liposomes, through sustained release and targeted delivery, are better suited for the prolonged action of anti-aging ingredients and efficient delivery of whitening agents to melanocytes [139,140,141,142]. For acne and hair loss, surface-modified polymeric carriers and vesicular systems enable follicular targeting and controlled release of antimicrobial agents [143]. Regarding soothing and repairing sensitive skin, polymeric micelles and functionalized nanoemulsions are ideal choices due to their excellent biocompatibility and ability to encapsulate anti-inflammatory ingredients [144]. Therefore, the rational selection of a carrier platform should directly correspond to the specific skin target and the desired clinical endpoint, thereby achieving an optimal balance of efficacy, safety, and formulation stability.

## 4. Safety and Regulation of Nanocarriers in Skincare

As the use of nanocarrier-based approaches for developing cosmetics is in full stride, it is important to ascertain the short-term and long-term safety of the products in terms of both consumer safety and environmental safety.

Owing to their minute sizes and unique shapes, nanoparticles can easily cross membranes, entering cells, tissues, and organs, and cause cell damage. Accordingly, the chemical reactivities and biological activities are usually greater at the nanoscale size as compared to the larger particles. This can lead to the increased generation of ROS and free radicals, which can bring about inflammation and induce oxidative stress, resulting in damage to cell membranes, proteins, and DNA [145]. For example, it was demonstrated that ZnO and TiO_2_ metal-containing nanocarriers can result in an increase in the production of cytokines and other pro-inflammatory mediators, thereby causing necrosis and allergies [16,146]. Thus, toxicity studies must be tailored to the specific properties of nanomaterials to ensure robust toxicity assessments. With regard to safety, regulations are defined as minimum mandatory criteria to obtain quality and safety. Due to the continuous use of nanotechnology in the cosmetic industry, regulatory bodies have been established around the globe.

In the U.S. cosmetic industry, regulatory efforts began with the Nanotechnology Task Force’s 2007 recommendations, which addressed concerns regarding nanostructure use in cosmetics. This was followed by the FDA’s 2011 publication of “Guidance for Industry: Safety of Nanomaterials in Cosmetic Products”, which is the first formal guidance document on the topic [147]. These considerations will be followed broadly for all FDA-regulated products, including cosmetic products, as their constituent particles belong to the nano range scale.

In the European Union, under Regulation (EC) No 1223/2009, the safety assessment of nanomaterials in cosmetics must be based on their intrinsic physicochemical characteristics and required toxicological data. The regulation stipulates that all ingredients and nanomaterials used in skincare products must undergo a safety assessment and be notified six months before marketing. Moreover, animal testing is strictly prohibited for the collection of toxicological information and hazard determination. Therefore, safety assessment of nanomaterials and nanocarriers is based on ex vivo and in vitro immunotoxicity tests [148].

It must be clarified that the foundational studies cited in this review, which may include data from animal experiments, are referenced solely to elucidate the fundamental transport pathways, biodistribution patterns, or proof-of-concept efficacy of nanocarriers. While such research has historically contributed to the scientific understanding of skin–barrier interactions, this citation in no way represents an endorsement or recommendation of animal testing for the development of modern cosmetic products. This position aligns fully with the increasingly stringent ethical and regulatory trends in major global markets.

For cosmetic companies to legally commercialize nanocarrier-based products, several compliant pathways exist. First, many core materials used to formulate nanocarriers may have existing safety profiles based on historical data or qualify for exemptions under recognized frameworks. The primary route for new ingredients, however, lies in the adoption of advanced non-animal testing methodologies. The field is increasingly reliant on a robust suite of New Approach Methodologies (NAMs) [149,150], including in vitro 3D reconstructed human skin models for assessing irritation, corrosion, and permeation; ex vivo human skin explants for more physiologically relevant penetration and metabolism studies; and computational (in silico) toxicology models for predicting toxicity endpoints [151].

Ultimately, the future of nanocarrier safety evaluation depends on the continued development, regulatory validation, and harmonized acceptance of these integrated testing strategies, ensuring both consumer safety and alignment with ethical imperatives.

## 5. Conclusions

In this review, we delve into the recent advances in nanocarrier-based topical delivery technology, providing a comprehensive overview of their principles, mechanisms, and diverse applications in skincare. By carefully selecting appropriate nanocarriers, this technology has significantly enhanced the permeation, stability, and bioavailability of active ingredients, thereby improving their therapeutic and cosmetic efficacy. In addition, we have systemically analyzed the advantages, challenges and practical considerations of different topical delivery systems, such as vesicular nanocarriers, lipid-based nanocarriers, and polymeric nanoparticles. Furthermore, we have explored the mechanisms of action of various topical technologies. Looking ahead, as the global emphasis on skin health, personalized care and aesthetic enhancement continues to grow, the application of topical delivery in cosmetics offers tremendous potential for addressing concerns such as anti-aging, skin brightening, moisturizing, and barrier repair. However, despite the substantial progress achieved, several challenges remain, particularly in ensuring long-term safety, scalability of production, controlled release, and compatibility with complex cosmetic formulations. Future research should prioritize the optimization of nanocarrier design, the integration of multifunctional materials, and the development of advanced formulation and evaluation techniques to ensure both efficacy and dermatological safety. Continued innovation in these areas will pave the way for modern topical cosmetic technologies, enabling more efficient, targeted, and safe delivery of active ingredients through the skin, which ultimately advances the field of skincare to benefit more individuals seeking skin health.

## Figures and Tables

**Table 1:** Comparison of advantages and limitations of different types of nanocarriers for skin delivery.

Nanocarriers	Advantages	Limitations	Ref.
Vesicular Nanocarriers (e.g., Liposomes, Transfersomes, Ethosomes, and Niosomes)	Excellent biocompatibility; Ability to encapsulate both hydrophilic and lipophilic agents; Biomimetic structure that facilitates fusion with skin	Limited physicochemical stability for some types; Topical delivery efficiency is highly dependent on composition and preparation methods	[54,55]
Lipid-Based Nanocarriers (e.g., (SLNs, Nanostructured lipid carriers (NLCs)))	Good biodegradability and safety; Solid matrix provides controlled release and protection; Forms an occlusive film to enhance skin hydration	SLNs may have limited drug loading and drug expulsion during storage; Production requires precise control to obtain stable nano-dispersions	[56,57]
Emulsion-Based Nanocarriers (e.g., Microemulsions, Nanoemulsions)	Relatively simple preparation; High loading capacity for hydrophobic actives; Ease of scale-up; Significantly improves skin hydration and penetration	High surfactant/cosurfactant concentrations may cause skin irritation; Long-term physical stability can be challenging.	[58,59,60]
Polymeric Nanocarriers (e.g., Polymeric nanoparticle (PNP), Polymeric micelle (PM), and Dendrimer)	Diverse structures with tunable properties (size, release profile, surface functionality); Enables sustained or stimuli-responsive release; Protects actives from degradation	Biocompatibility and degradability of some synthetic polymers require careful assessment; Potential toxicity of some materials or degradation products	[61,62]
Inorganic Nanoparticles (e.g., Mesoporous Silica, Titanium Dioxide, Carbon, and Metallic nanomaterials)	High stability; Unique optical, electrical, or catalytic properties; Mesoporous materials offer high surface area and pore volume for high drug loading	Long-term dermal safety profile requires comprehensive evaluation; Potential environmental accumulation risks	[63,64]
Inclusion Complexes (e.g., Cyclodextrins (CDs))	Significantly improves solubility and stability of poorly soluble actives; Reduces irritation and volatility of actives; Enhances formulation compatibility	Primarily suitable for small molecules; Limited loading capacity; Penetration enhancement effect on the skin barrier is generally indirect and mild	[65,66]

**Table 2:** Mechanisms of action and representative ingredients of freckle-removal and whitening cosmetics.

Basic Mechanics	Mechanism of Action	Pathway of Action	Representative Ingredients	Mechanism of Skin Lightening and Spot Removal	Ref.
Antagonistic Agents	Suppress the secretion of melanocyte-stimulating hormones induced by external stimuli such as sunlight and stress	It contains substances that compete with melanin-stimulating hormones, blocking the signaling pathway	Undecylenoyl phenylalanine	Melanin antagonist, inhibiting melanin formation signals and subsequently suppressing melanogenesis	[88,89]
Inhibitory Agents	Highly skin-penetrating ingredients that reach the dermis layer, inhibiting melanin production and oxidation during its formation phase	By inhibiting tyrosinase activity through mechanisms such as preventing substrate–enzyme binding and tyrosinase–substrate complex formation, as well as suppressing oxidative processes	Licorice glabra root extract, arbutin and its derivatives	By inhibiting the activity of tyrosinase, the production of melanin is prevented	[90,91,92,93]
			Vitamin C and its derivatives	By inhibiting the catalytic reaction of tyrosinase, which reduces the intermediate dopachrome in the melanin synthesis pathway, thereby suppressing melanin production	
Blocking Agents	During the melanin transfer phase, the delivery speed of melanosomes to keratinocytes is reduced, leading to a decrease in melanin content across various layers of epidermal cells	By reducing the activity of melanosomes, the melanin content within cellular layers is decreased	Nicotinamide	It can act on already formed melanin, reducing its transfer to superficial cells	[94]
Metabolic Agents	It softens keratin, accelerates the shedding of dead cells, and promotes epidermal metabolism, allowing melanosomes that have entered the epidermis to be shed along with the rapid renewal of the epidermis during the metabolic process	Employing chemical or biological exfoliants to sweep away the superficial keratinocytes that contain melanin	Alpha hydroxy acids, keratolytic enzymes	After penetrating into the SC of the skin, it accelerates the rate of cell renewal and promotes the shedding of dead cells, thereby improving skin condition	[95,96]

## Data Availability

No new data were created or analyzed in this study. Data sharing is not applicable to this article.

## Abbreviations

SC	Stratum corneum
SLNs	Solid lipid nanoparticles
EE	encapsulation efficiency
NLCs	Nanostructured lipid carriers
PNP	Polymeric nanoparticle
PM	Polymeric micelle
CDs	Cyclodextrins
UV	Ultraviolet
CPDs	Cyclobutane pyrimidine dimers
NEG	non-enzymatic glycation
PPE	Porcine placenta extract
ROS	Reactive oxygen species
RT	Retinol palmitate
EGCG	Epigallocatechin gallate
FA	Ferulic acid
T-Res	Trans-Resveratrol
CUR	Curcumin
FDA	United States Food and Drug Administration
Ga	*Gelidium amansii*
UP	Undecylenoyl phenylalanine
HA	hyaluronic acid
LMW-HA	low molecular weight HA
NAMs	New Approach Methodologies
